# Evaluation of neutrophile-to-lymphocyte ratio and lipid profile in non-violent suicide attempters: a mechanistic study

**Published:** 2024-07

**Authors:** Sajjad Salari, Maryam Bagheri

**Affiliations:** ^*a*^ Department of Physiology, Faculty of Medicine, Ilam University of Medical Sciences, Ilam, Iran.

**Keywords:** Triglyceride, Total cholesterol, LDL, HDL, NLR, Suicide attempt

## Abstract

**Background:**

Suicide is one of the most common causes of juvenile death. Screening suicide risk is quite challenging and even more difficult in subjects who have no psychiatric disorder or other suicide risk factors in their medical history. To date, the association of serum lipid profile and suicidal risk has been evaluated in patients with different psychiatric disorders, yielding conflicting results. Here, we aimed to investigate the lipid panel and neutrophil-to-lymphocyte ratio in the sera of people with a first episode of suicide attempt in western Iran.

**Methods:**

A total of 159 suicide attempters and 186 volunteers without history of suicide, aged 18-35 years, were evaluated in this experiment. Blood samples were collected between 8-10 AM and kept at 37 °C for blood clotting. We then analyzed the concentration of various lipid markers, including triglyceride (TG), total cholesterol (TC), low-density lipoprotein (LDL), and high-density lipoprotein (HDL), using a series of enzymatic reactions. Additionally, we determined the neutrophil-to-lymphocyte ratio (NLR) by dividing the total number of neutrophils by the total number of lymphocytes after conducting peripheral blood cell counts.

**Results:**

The concentration of TG was 94.2±5.8 mg/dL in males and 92.3±5.3 mg/dL in females. TC was 136.3±3.6 mg/dL in males and 152.7±4.4 in females, with a significant decrease in comparison with the control subjects (p less than 0.0001). NLR was 4.34±0.9 with a significant increase compared with the controls (P less than 0.002). (TG), (TC) and (LDL) levels in suicide attempters were significantly lower than in the control groups. Nevertheless, serum (HDL) levels in male and female suicide attempters were significantly higher than in the control group. Furthermore, NLR was significantly lower in the subjects with suicide attempts compared with the controls.

**Conclusions:**

Lower concentrations of (TG), (TC) and (LDL) along with higher NLR were associated with non-violent suicide attempts. These findings might be an effective tool in screening suicide risk in young adults.

## Introduction

The World Health Organization (WHO) estimated that the global suicide rate is approximately 9 cases per 100,000 individuals, with a higher prevalence in low and middle-income countries.^1^ While complete suicides are more common among the elderly, there has been a notable increase in suicide attempts among young adults in recent decades.^1-3^ In a 5-year study Ilam province, western Iran, the incidence of suicide attempts in men was reported to range from 172.2 to 177.9 per 100,000 populations per year, while in women, the range was from 144.9 to 221.1.^4^ Neuropsychiatric disorders, alcoholism, drug abuse, and previous suicide attempts are some of the suicidal behavior risk factors,^1^ which might help screen susceptible subjects for upcoming suicide attempts. Notably, there is still no effective approach to identifying suicidality in people without any confirmed psychiatric disorder and/or other associated individual factors. Recently, we have found an increased level of oxidative stress in young subjects with the first episode of suicide attempts^2^ that confirmed the benefits of neurobiological factors in screening suicidal behavior. Biological markers, particularly endocrine measurements, are increasingly being integrated into clinical psychological research. peripheral cortisol concentrations as a biomarker for post-traumatic stress disorder (PTSD)^3^ have been approved in a study. Serotonin hypofunction has been found in suicide and suicide attempts in different in vivo and postmortem studies.^4^ Increased serum levels of GFAP and NSE have been reported recently in subjects with suicide attempts.^5^ Serum lipids are another biological factor that is measured routinely in clinics. Much study has been conducted on lipid profiles in suicide attempt behavior. The concentration of (TG), (TC), HDL-C, and LDL-C have exhibited alterations in individuals who have attempted suicide and concurrently present with psychiatric disorders.^6-9^ Decreased serum content of (TC), (LDL) and TG have been detected in suicide attempts in affected roles with major depressive disorder (MDD).^7,10,11^ Low serum (TC) is also reported in suicide attempted-subjects with different psychotic syndromes and major depressive disorder.^6,12^ Furthermore, the presence of low serum (TC) was correlated with violence in these patients.^6^ On the contrary, in a study by Shakeri et al. high serum (TC) levels have been detected in bipolar patients with suicide attempts.^13^

Accordingly, our objective was to scrutinize alterations in the serum lipid panel besides the neutrophil-to-lymphocyte ratio among individuals experiencing their initial episode of non-violent suicide attempt, devoid of documented psychiatric illnesses or indications of suicidality in their medical records, within the region of Western Iran. 

## Methods 

**Subjects**

159 (93 men and 66 women) non-violent suicide attempters and 186 (102 men and 84 women) volunteers without a history of suicide aged 25 to 35 years were evaluated in this research. Suicide-attempted subjects were admitted at the Shahid Mostafa Khomeini Hospital from March 2017 to March 2019.

All subjects signed the written informed consent before the experiment. A survey was employed to gather the demographic items. IR.MEDILAM.REC.1398.165 is the ethics committee-approved ID.

**Inclusion/ Exclusion criteria**

Subjects with the first episode of suicide attempt, aged 25-35, using non-violent suicide methods were included in the study.

People with a history of drug and alcohol abuse, confirmed psychiatric illness (on medication), diabetes mellitus, cardiovascular disease, liver disease, and thyroid hormone disturbance, and subjects using cholesterol-lowering and anticoagulant agents were excluded from the study. 

The blood samples were collected from all subjects between 8-10 AM during the first 24 hours after admission. Some were transferred into heparinized containers for NLR measurement. The rest were kept for 10 minutes at 37 °C for blood clotting. Ultimately, the samples underwent centrifugation at 3500 (rpm) for 10 minutes. Subsequently, the resultant supernatant serum was carefully extracted and stored at -80°C. Serum triglyceride levels were measured using a series of enzymatic reactions. Firstly, the triglycerides are hydrolyzed to glycerol by lipase. Then, glycerol was phosphorylated and then oxidized. Finally, H2O2, which is produced as a byproduct, is reacted with 4-aminophenazone and phenol under a peroxidase enzyme to produce color development and absorbance is measured at 500 nm.

Total serum cholesterol level was measured as well. Cholesterol esters were first hydrolyzed by cholesterol ester hydrolase then cholesterol oxidized by the oxidase enzyme at the 3-OH group position. H2O2, which is produced as a byproduct in this reaction, is reacted with peroxidase enzyme 4-aminophenazone and phenol and induces color development whose absorbance is measured at 500 nm.

To quantify HDL cholesterol, the esters within HDL were initially subjected to hydrolysis, initiating a process of oxidation specifically targeting HDL cholesterol, which led to the production of hydrogen peroxide (H2O2) as a secondary product. This ensuing H2O2 was then engaged in a chemical reaction with 4-aminophenazone and N-Ethyl-N-(3-methylphenyl)-N'succinyl ethylene diamine, catalyzed by the peroxidase. The resulting reaction product was subsequently measured for its absorbance at a wavelength of 600 nm.

LDL cholesterol is calculated based on the following formula;

LDL-cholesterol = [total cholesterol] - [HDL-cholesterol] - [TG] / 5.

NLR was assessed by dividing the aggregate count of neutrophils by the total count of lymphocytes, utilizing peripheral blood cell enumeration.

**Statistical analysis**

The data analysis was conducted using Graph Pad Prism 8 software. Statistical comparisons of lipid profile parameters between groups were carried out using one-way ANOVA and Mann-Whitney tests. A significance level of P <0.05 was considered significant for the analysis.

## Results

In the control group, the mean age was 33.3±0.2 years for men and 33.7±0.16 years for women. The mean age of male suicide attempters was 33.7±1.35, and of female suicide attempters was 32.74±1.6 years.

**Figure 1:**
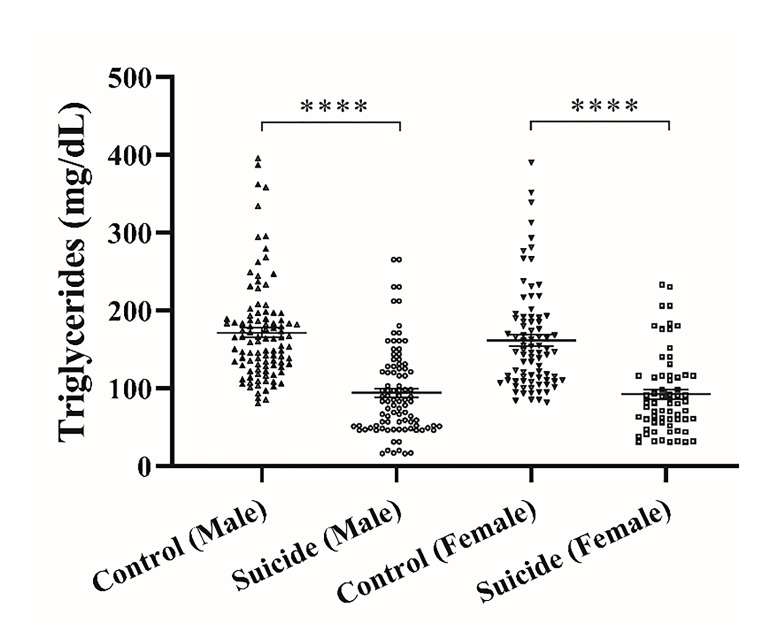
Serum triglyceride levels in suicide attempters and control groups. The graph represents Mean ± SEM in the different groups. * Indicates a significant difference between the suicide attempters and control groups; ****P <0.0001.

Figure 1 shows serum (TG) levels in suicide attempters and control groups. Serum (TG) levels in male and female control subjects were 171.6±6.4 and 161.6±7.1 mg/dL correspondingly. Serum (TG) levels in male and female suicide attempters were 94.2±5.8 and 92.3±5.3 mg/dL, respectively, which were significantly lower than controls (P<0.0001). There was not any significant difference between male and female intergroups. 

**Figure 2:**
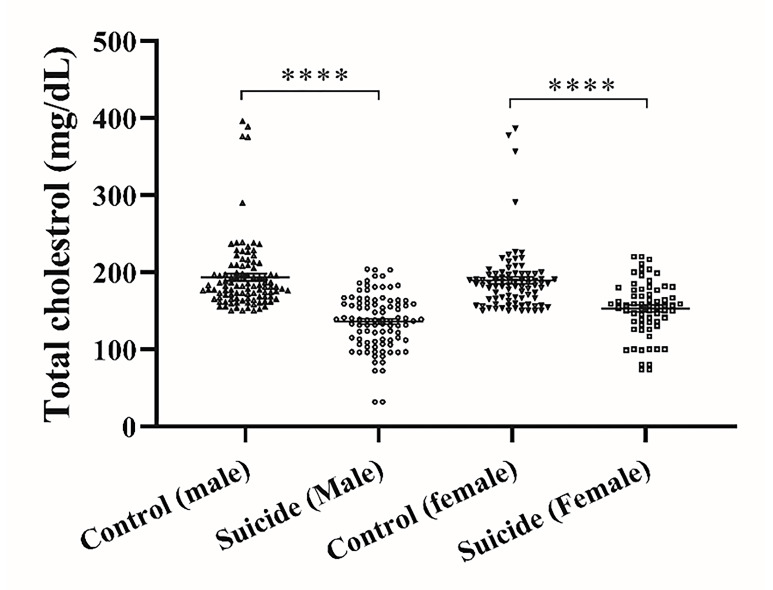
Serum total cholesterol levels in suicide attempters and control groups. The graph represents Mean ± SEM in the different groups. * a significant difference between the suicide attempters and control groups; ****P <0.0001.

Figure 2 depicts the serum (TC) levels in both suicide attempters and control groups. Among control subjects, male and female individuals exhibited serum TC levels of 193.7±4.5 mg/dL and 189.3±4.6 mg/dL, correspondingly. In contrast, male and female suicidal subjects displayed notably lower serum TC levels, measuring 136.3±3.6 mg/dL and 152.7±4.4 mg/dL, respectively. These observed TC levels were significantly diminished in comparison to the control counterparts (P<0.0001). Notably, there was a significant difference in TC serum levels in male vs. female suicide attempters (P<0.005). 

 shows serum LDL levels in suicide attempters and control groups. Serum LDL levels in male and female control subjects were 105.9±0.87 and 106.8±0.87 mg/dL, respectively. This parameter in male and female suicide attempters was 75.7±3 and 80±3.1 mg/dL, respectively, which were significantly lower than these values in control groups (P<0.0001). This value did not change significantly in the male vs. female intergroup.

**Figure 3:**
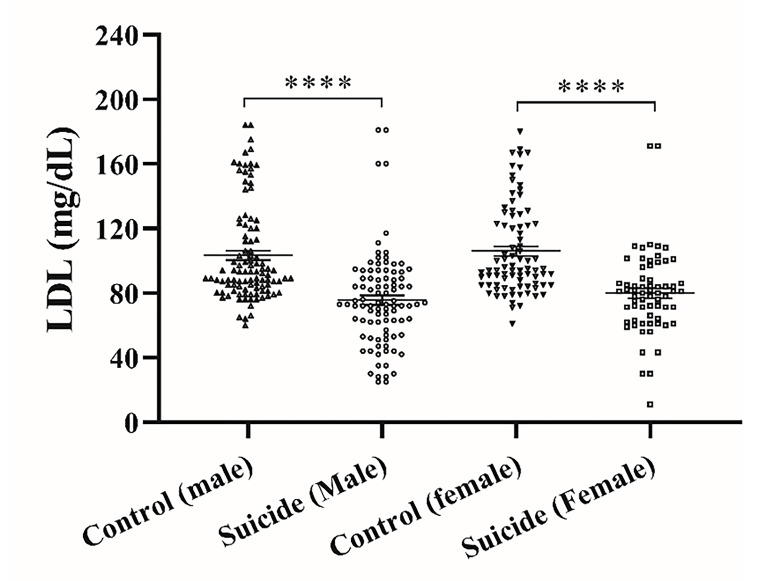
Serum LDL-cholesterol levels in suicide attempters and control groups. The graph represents Mean ± SEM in the different groups. * Indicates a significant difference between the suicide attempters and control groups; ****P <0.0001.

**Figure 4:**
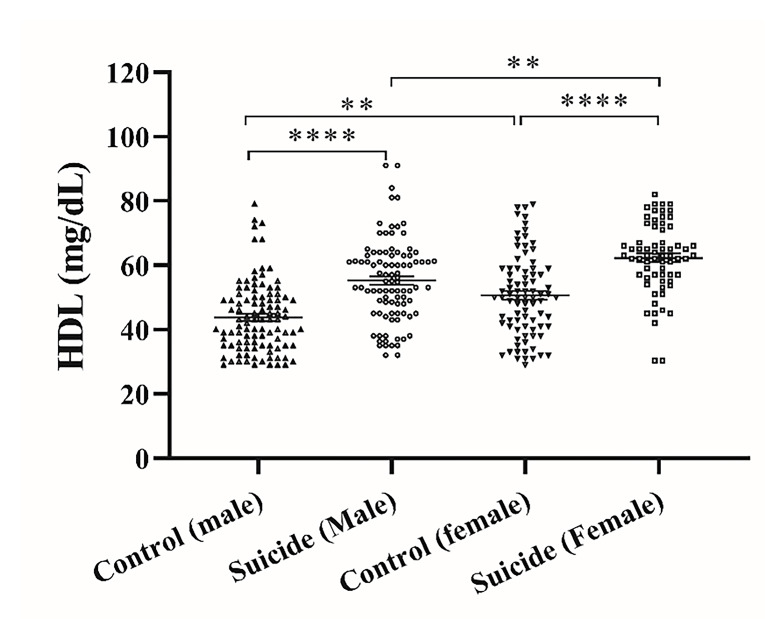
Serum HDL-cholesterol levels in suicide attempters and control groups. The graph represents Mean ± SEM in the different groups. *a significant difference between the suicide attempters and control groups; (**P <0.01 and ****P <0.0001).

Serum HDL levels in suicide attempters and control groups are exhibited in Figure 4 . Serum HDL level in the male control subjects was 43.1±0.32 mg/dL and in the male suicide attempters was 55.51±1.3 mg/dL which showed a significant increase in the latter (P<0.0001). Furthermore, this value was 51.15±0.39 mg/dL in the female control subjects while it was significantly changed to 74.73±1.3 mg/dL in the female suicide attempters (P<0.0001). Notably, there was a significant difference in the serum (HDL) level in male and female suicide attempters and in male and female controls (P<0.0001 and P<0.001 respectively).

NLR was 4.34 ± 0.9 in the non-violent suicide attempted group and 2.37± 1.1 in the controls which showed a significant increase in non-violent suicide attempts (Table 1 ; P<0.002). 

**Table 1:** Neutrophil to lymphocyte ratio (NLR) in all subjects

Neutrophil to lymphocyte ratio (Mean ± SEM)		
non-violent SA (n=159)	Controls (n= 186)	P-value
4.34 ± 0.9	2.37± 1.1	P<0.002

NLR is increased significantly in non-violent suicide attempters compared with the controls (P<0.002). SA; suicide attempt.

## Discussion

The outcomes of our investigation revealed diminished concentrations of (TG), (TC), and (LDL), coupled with elevated levels of (HDL) within both male and female individuals who had engaged in non-violent suicide attempts in contrast to corresponding subjects devoid of any such history. Additionally, the neutrophil-to-lymphocyte ratio (NLR) exhibited a notable elevation among individuals with a history of suicide attempts compared to the control group.

Prior investigations have scrutinized the correlation between serum total (TC) levels and the propensity for suicide. For instance, Bondoc et al. (2019) conducted an assessment of serum (TC) levels among Romanian mothers who had experienced suicide attempts during the postpartum phase. In congruence with our observations, their findings revealed diminished serum (TC) levels among those with a history of suicide attempts in comparison to control subjects.^14^ The relationship between decreased serum (TC) levels and the vulnerability to suicide has also been substantiated among young Japanese employees. Studies have indicated that for every 10 mg/dL reduction in average TC levels, there is an associated 18% rise in the likelihood of death by suicide.^15^ Despite the absence of documented psychiatric disorders in the medical histories of the individuals who engaged in suicide attempts within the scope of the present investigation, it remains plausible that the incipient stages of (MDD) were not entirely excluded in these subjects. A contemporary study delved into the serum lipid profiles of individuals diagnosed with MDD who experienced their inaugural episode of suicide attempt. While their findings delineated elevated levels of (LDL) and reduced levels of (HDL) within the cohort exhibiting suicide attempts, opposite to our results, a concurrence was observed regarding diminished serum (TC) levels among suicide attempters when compared with the controls.^7^ It is imperative to note the existing controversy surrounding the correlation between serum lipid profiles and the propensity for suicide within the context of (MDD) patients. Lee et al. (2003) documented decreased serum levels of (TG), (TC), and (LDL-C) among Korean individuals diagnosed with MDD who had attempted suicide, in contrast to MDD patients devoid of any history of suicide attempts.^16^ Even though Spanish researchers in 2007 confirmed decreased levels of (TG), (TC) and LDL-C in male MDD suicide attempters compared to controls; however, they found no significant changes in females.^17^ Baek et al. in 2014 indicated low serum TG content and high HDL-C in US (MDD) patients with suicide attempts; however, they found no significant difference in LDL-C level.^11^ It should be noted that the serum value of TG and LDL did not significantly change in male and female control and suicide attempted groups. Except for TC in suicide-attempted subjects which changed significantly between males and females in the suicide group. Notably, lower NLR was reported in a study on violent suicide attempters in Italy.^18^ However, another study from Spain released a higher NLR ratio in suicide attempted subjects who have lower depression severity.^19^ Although, our results showed a higher NLR ratio in non-violent suicide attempts. Nevertheless, most of the studies propose further research into lipid and NLR roles in suicide attempt risk.

Notably, brain neurons don’t have access to blood lipoproteins, due to blood-brain barrier (BBB) prevention, and it is difficult to interpret the correlation of serum lipid profile and suicidal behavior. However, higher suicide risk has been indicated in people carrying gene variants that impair cholesterol synthesis.^20-22^ It has been indicated that a small reduction in cholesterol/phospholipid ratio in synapses lipid raft can impair GABA and serotonin transmission in the brain. Both GABA and serotonin hypofunction have been linked to impulsivity, aggression, and suicide behavior.^23-25^ Contrary, higher NLR might indicate the presence of inflammation,^26^ as evidenced by our recent discovery of neuroinflammation in suicide-attempted subjects while lower NLR might indicate a more robust immune response.^27^

This study concluded that lower serum levels of triglycerides (TG), total cholesterol (TC), and low-density lipoprotein (LDL), coupled with a higher neutrophil-to-lymphocyte ratio (NLR), were correlated with non-violent suicide attempts in Ilam province. This suggests the potential use of these biomarkers as effective tools for screening suicide risk in young adults. The findings of this study are expected to pave the way for further research into the relationship between lipid profiles and NLR in individuals at higher risk of suicide. It is worth noting that serum lipid profiles and NLR might serve as valuable indicators for understanding suicidal behavior within the population.

**Limitations of the study**

Interviewing individuals who have attempted suicide can be challenging due to the sensitive nature of suicidal behavior in Iranian society. Additionally, the documentation of medical cases involving individuals susceptible to suicide often lacks robustness. It would be beneficial to have access to the lipid profile and NLR levels of subjects at least six months before their suicide attempt. This data could potentially provide valuable insights into the underlying mechanisms of such behavior.

**Acknowledgement**

Our thanks go to Ilam University of Medical Sciences and Shaheed Mostafa Khomeini University Hospital for their support during achievement of this study. 
